# Molecular Action of Polyphenols in Leukaemia and Their Therapeutic Potential

**DOI:** 10.3390/ijms22063085

**Published:** 2021-03-17

**Authors:** Hamza A. Alaswad, Amani A. Mahbub, Christine L. Le Maitre, Nicola Jordan-Mahy

**Affiliations:** 1 Biomolecular Sciences Research Centre, Department of Biosciences and Chemistry, Sheffield Hallam University, The Owen Building, City Campus, Howard Street, Sheffield S1 1WB, UK; h.alaswad@shu.ac.uk (H.A.A.); hwbcl3@exchange.shu.ac.uk (C.L.L.M.); 2 Laboratory Medicine Department, Faculty of Applied Medical Sciences, Umm Al-Qura University, P.O. Box 715, Makkah 21955, Saudi Arabia; aamahbob@uqu.edu.sa

**Keywords:** polyphenols, leukaemia, reactive oxygen species, glutathione, cell cycle, apoptosis, autophagy, multi-drug resistance

## Abstract

Leukaemia is a malignant disease of the blood. Current treatments for leukaemia are associated with serious side-effects. Plant-derived polyphenols have been identified as potent anti-cancer agents and have been shown to work synergistically with standard chemotherapy agents in leukaemia cell lines. Polyphenols have multiple mechanisms of action and have been reported to decrease cell proliferation, arrest cell cycle and induce apoptosis via the activation of caspase (3, 8 and 9); the loss of mitochondrial membrane potential and the release of cytochrome c. Polyphenols have been shown to suppress activation of transcription factors, including NF-kB and STAT3. Furthermore, polyphenols have pro-oxidant properties, with increasing evidence that polyphenols inhibit the antioxidant activity of glutathione, causing oxidative DNA damage. Polyphenols also induce autophagy-driven cancer cell death and regulate multidrug resistance proteins, and thus may be able to reverse resistance to chemotherapy agents. This review examines the molecular mechanism of action of polyphenols and discusses their potential therapeutic targets. Here, we discuss the pharmacological properties of polyphenols, including their anti-inflammatory, antioxidant, anti-proliferative, and anti-tumour activities, and suggest that polyphenols are potent natural agents that can be useful therapeutically; and discuss why data on bioavailability, toxicity and metabolism are essential to evaluate their clinical use.

## 1. Introduction

### 1.1. Leukaemia

Leukaemia is described as a heterogeneous group of haematological cancers [1,2], defined as a malignant disease of the blood, characterised by uncontrolled proliferation and the development of leucocytes in the blood [3] and bone marrow [4] that accumulate in the liver and spleen [1].

Since its original identification by Virchow in 1847 [5], the general classification of leukaemia has become more complex [1]. At present, leukaemia is classified into two main types: myeloid or lymphoid, based on the predominant cell of origin and is sub-divided into acute or chronic, based on the rate of progression [6] with multiple sub-classifications within each group [7].

The current therapies for leukaemia include chemotherapy, radiotherapy, immuno-therapy, and bone marrow transplantation [6,8]. Chemotherapy remains the front-line treatment for most leukaemias [9]. Leukaemia chemotherapies include anti-metabolites (e.g., methotrexate) [10,11]; topoisomerase II inhibitors (e.g., doxorubicin) and alkaloids (e.g., omacetaxine) [12]. In addition to the general chemotherapy agents, targeted therapies are used for specific leukaemias, such as tyrosine kinase inhibitors (e.g., Imatinib), which are used for BCR/ABL positive leukaemia [13,14]. However, many chemotherapy agents are associated with serious side effects, such as liver damage, nerve damage, heart disorders and loss of immunity [6], which result in poor adherence to treatment regimens and poor prognosis [6]. Thus, new treatments for leukaemia are needed, which could be used individually or in combination with existing standard chemotherapy agents, in an attempt to potentiate anti-tumour effects, whilst reducing treatment doses and side effects. One potential source of therapeutics is polyphenols.

### 1.2. Polyphenols

**Figure 1:**
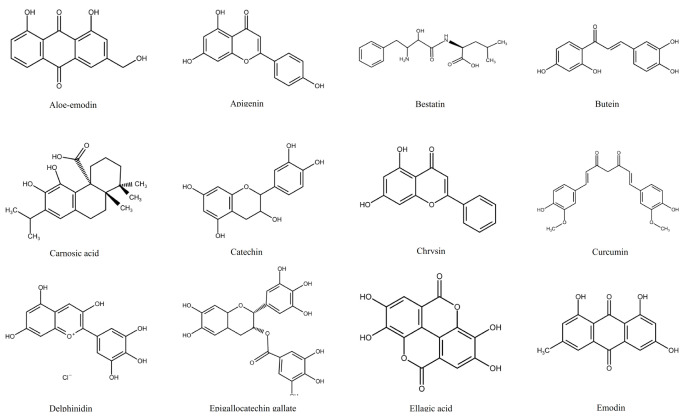

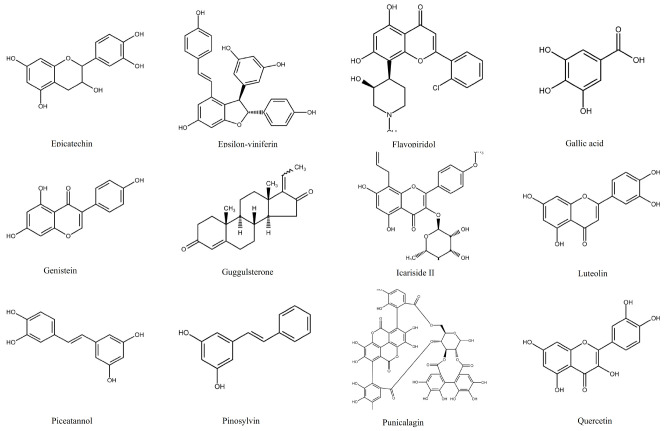

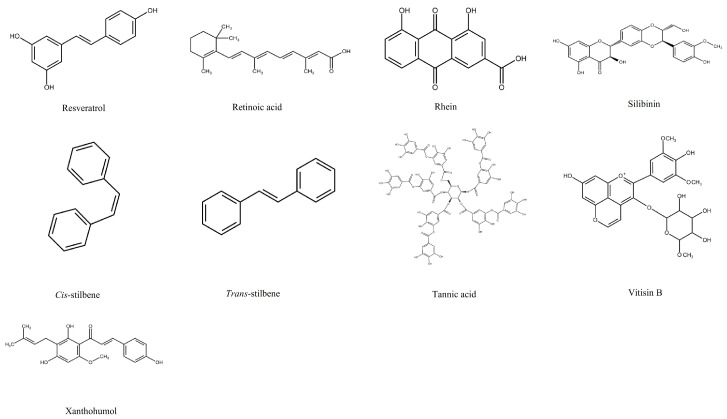
Chemical structures of all polyphenols discussed within this review. Aloe-emodin [29], Apigenin [30], Bestatin [31], Butein [32], Carnosic acid [33], Catechin [34], Chrysin [35], Curcumin [36], Delphinidin [37], Epigallocatechin gallate [38], Ellagic acid [39], Emodin [40], Epicatechin [41], Epsilon-viniferin [42], Flavopiridol [43], Gallic acid [44], Genistein [45], Guggulsterone [46], Icariside II [47], Luteolin [48], Piceatannol [49], Pinosylvin [50], Punicalagin [51], Quercetin [50], Resveratrol [52], Retinoic acid [53], Rhein [54], Silibinin [55], *Cis*-Stilbene [56], *Trans*-Stilbene [50], Tannic acid [57], Vitisin B [58], Xanthohumol [59].

Polyphenols are a family of phenolic phytochemicals [15] (Figure 1). They are an essential part of the diet, and are found in fruits, vegetables, cereals, nuts, and herbs [16], and drinks such as wine, beer, tea, and coffee [17]. Epidemiological and animal studies have shown potential benefits, with protective effects seen against a wide range of conditions, including cancers such as leukaemia [17,18]. Polyphenols’ anti-cancer activities include halting cell cycle, inducing apoptosis, modulation of angiogenesis pathways, and preventing metastasis [19]. Studies have shown that the consumption of polyphenol-rich foods can prevent 10 to 70% of cancer deaths [18,19]. A major advantage of the use of polyphenols as anti-cancer agents, is that they have low toxicity, and are safe to consume, and highly accessible [20]. This could offer opportunities for innovation in drug discovery [21] and could play a major role in cancer prevention [22]. Moreover, a number of polyphenols (e.g., quercetin, apigenin, rhein, emodin and resveratrol) have been shown to act synergistically with chemotherapy agents when used as combination treatments in vitro, enhancing cell cycle arrest and inducing apoptosis [23,24,25]. These synergistic effects were shown to be at least partly regulated by a decrease in glutathione levels and an increase in DNA damage, when polyphenols were combined with etoposide; doxorubicin [24,25,26]; cisplatin and 5-fluorouracil in leukaemia cell lines [24,26,27,28].

Here, we summarise the molecular actions of polyphenols and discuss their potential therapeutic use. We outline how they act at a cellular and molecular level in a wide variety of cell lines (Table 1) which represent the different forms of leukaemia, and ask the question: Is there sufficient evidence to progress towards clinical trials for polyphenols as an adjunctive treatment alongside standard chemotherapy agents?

## 2. Molecular Mechanisms of Polyphenols

**Figure 2:**
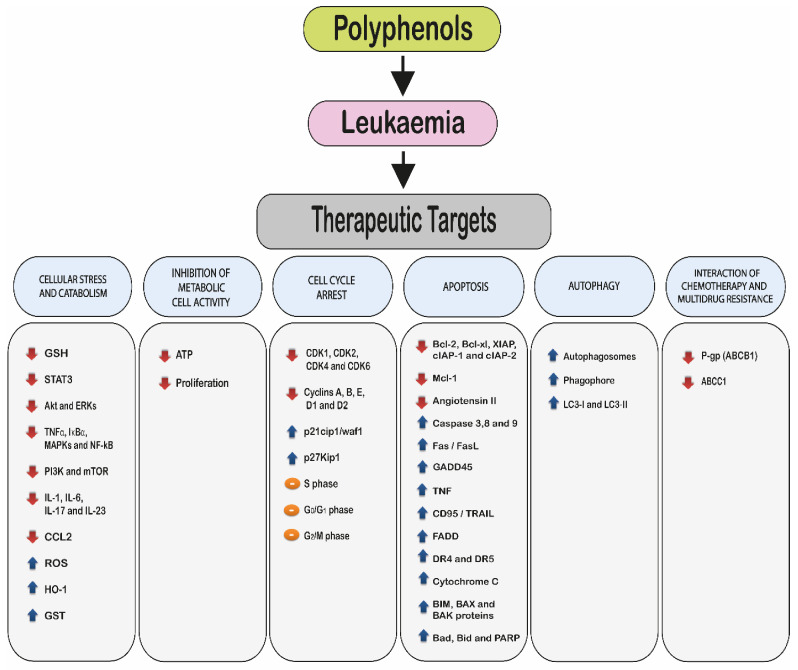
Molecular targets of polyphenols in leukaemia. The arrows represent changes in the levels and activities of genes and proteins. The red arrow shows an inhibition or reduction in gene expression and/or protein production, whilst the blue arrow shows an induction or increase gene expression and protein production. The orange sign indicates cell cycle arrest. The figure does not indicate any hierarchy.

Polyphenols target several molecular pathways leading to cell cycle arrest and induced apoptosis in leukaemia cell lines, while being protective to non-tumour control cells [67,68,154,155] (Figure 2). The key molecular targets of polyphenols include:

2.1. Induction of cellular stress and catabolism: through an increase in reactive oxygen species (ROS) and a decrease in cellular antioxidants such as glutathione (GSH).
2.2. Modulation of cell metabolic activity.
2.3. Cell cycle arrest.
2.4. Induction of cell death via:
  - Apoptosis.
  - Autophagy.
2.5. Interaction with chemotherapy agents and the reduction or reversal of multidrug resistance.

These targets make polyphenols an attractive potential therapy for use in leukaemia.

Abbreviations: Glutathione (GSH), Signal transducer and activator of transcription 3 (STAT3), Protein kinase B (Akt), Extracellular signal-regulated kinases (ERKs), Tumour necrosis factor-α (TNFα), inhibitor of NF-κB (IκBα), Mitogen-activated protein kinase (MAPKs), Nuclear factor κB (NF-κB), Phosphoinositide 3-kinase (PI3K), Mammalian target of rapamycin (mTOR), Interleukin-1 (IL-1), Interleukin-6 (IL-6), Interleukin-17 (IL-17), Interleukin-23 (IL-23), CC-chemokine ligand 2 (CCL2), Reactive oxygen species (ROS), Heme oxygenase-1 (HO-1), Glutathione S-transferases (GST), Adenosine triphosphate (ATP), Cyclin dependent kinases 1 (CDK1), Cyclin dependent kinases 2 (CDK2), Cyclin dependent kinases 4 (CDK4), Cyclin dependent kinases 6 (CDK6), Cyclin-dependent Kinase Inhibitor (p21CIP/WAF), Cyclin-dependent kinase inhibitor (p27Kip1), mitogen-activated protein (MAP), B-cell lymphoma 2 protein (Bcl-2), B-cell lymphoma extra-large (Bcl-XL), X-linked inhibitor of apoptosis protein (XIAP), Cellular Inhibitor of Apoptosis Protein 1(cIAP-1), Cellular Inhibitor of Apoptosis Protein 2 (cIAP-2), Myeloid cell leukaemia 1 (Mcl-1), Cysteine aspartic acid specific protease 3, 8 and 9 (Caspase 3, 8 and 9), APO-l/CD95/tumour necrosis factor superfamily 6 (TNFRSF6)/APT-1(Fas), Fas Ligand (FasL), Tumour necrosis factor receptor (TNF), Fas-associated Protein with Death Domain (FADD), Death receptor 4 and 5 (DR4 and DR 5), Bcl-2-like protein (BIM), Bcl-2-associated X protein (Bax), Bcl2-antagonist/killer (Bak), the Bcl2 associated agonist of cell death (Bad), BH3 interacting-domain death agonist (Bid), Poly (ADP-ribose) polymerase (PARP), Growth Arrest and DNA Damage (GADD45), Light chain I and II (LC3-I and LC3-II), Permeability glycoprotein (P-gp), ATP Binding Cassette Subfamily C Member 1 (ABCC1).

### 2.1. Polyphenols, Cellular Stress and Catabolism

Inflammation is associated with the development and initiation of most cancers, by activating cellular signals that lead to DNA damage and several epigenetic changes [156]. This is accompanied by an increase in inflammatory cell infiltration and the activation of immune cell signalling factors such as nuclear factor κB (NF-κB) and STATs [156,157]. This leads to increased activation of chemokines, cyclooxygenase (COX)-2 enzyme, prostaglandin E2 (PGE2), inducible nitric oxide synthase (iNOS), matrix metalloproteinase 9 (MMP-9), vascular endothelial growth factor (VEGF), and cytokine production (e.g., interleukin (IL)-1, IL-6 and IL-8) [157,158,159]. The role of inflammation and cancer progression has been reviewed extensively [156,157,158,159]. Targeting these signalling pathways represents an attractive strategy for the prevention and treatment of cancer [156,157,158,159].

Upregulation of signal transducer and activator of transcription 3 (STAT3) and NF-κB are two fundamental transcription factors that are associated with many types of cancer, including leukaemia [159,160,161,162]. NF-κB can affect all six hallmarks of cancer through the transcriptional activation of more than 200 genes associated with cell proliferation, angiogenesis, metastasis, tumour promotion, inflammation and suppression of apoptosis [163,164,165]. One of the most documented functions of NF-κB is its ability to promote cell survival through the induction of target genes, the products of which inhibit the apoptotic machinery in both normal and malignant cells [166,167]. NF-κB can also prevent programmed necrosis by inducing genes that encode antioxidant proteins [167]. In most cases, NF-κB is maintained in an active state within cancer cells through mutational the activation of upstream signalling molecules or in response to extracellular stimuli within the tumour microenvironment [168].

**Figure 3:**
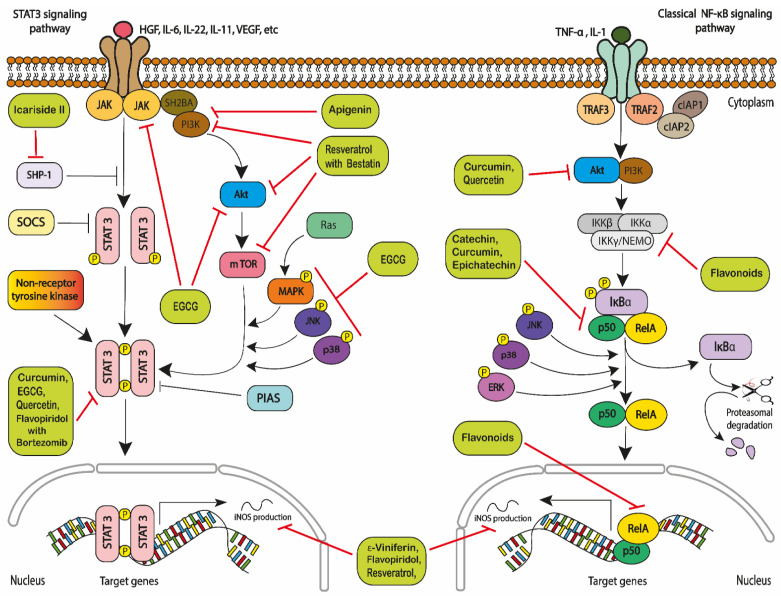
The classical NF-κB (**Right**) and STAT3 (**left**) activation pathways. The NF-κB pathway is initiated by numerous stimuli including TNFα and IL1 and is mediated by the IκB kinase (IKK) complex. This results in the phosphorylation of IkBα and leads to degradation by the proteasome. This releases RelA/p50 complex to be translocated into the nucleus and binds to DNA to induce the expression of specific genes. STAT3 is activated by the Janus kinase (JAK)/signal transducer, non-receptor tyrosine kinase signalling pathways and Ras/mitogen-activated protein kinase (MAPK). Suppressor of cytokine signalling (SOCS) and protein inhibitor of activated STAT3 (PIAS3) can negatively regulate the activity status of STAT3. The activated form of STAT3 translocate into the nucleus and binds to the DNA of specific genes.

This increase in the activation of NF-κB in cancer cells increases cell survival and proliferation; and increases expression of inflammatory cytokines and growth factors in leukaemia cells [161,169]. The activation of NF-kB can be mediated by either the canonical or the alternative pathway (Figure 3). The canonical pathway is mainly activated by extracellular factors such as ligands [170]. The activation of NF-κB is most commonly mediated by the IκB kinase (IKK) complex, which is composed of two catalytic subunits, IKKα and IKKβ, and a regulatory subunit IKKγ/NEMO [171,172]. IKK-mediated IκB phosphorylation leading to successive ubiquitination by the SCF–βTrCP complex and its degradation by the proteasome [173], this means that it subsequently releases NF-κB dimers, which enter the nucleus [172]. The NF-kB heterodimers including p50, p65, and/or RelA [170], which translocate into the nucleus where it becomes phosphorylated [171,174], and regulated by other post-translational modifications, such as protein acetylations [175]. Since RelA/p50 complex binds to DNA, it becomes able to induce the expression of specific genes, such as anti-apoptotic genes cIAP2, Bcl-2 and Bcl-xL [174] to stimulate cell proliferation [176,177] (Figure 3).

The canonical or classical pathway is the most widely known route to NF-κB activation. It is essentially mediated by the action of the RelA/p50 subunits, while the non-canonical or alternative pathway mainly activates RelB-p52 complexes through the inducible processing of p100 [178]. In contrast to the canonical pathway, this pathway is activated by a more restricted number of ligands, such as the B-cell-activating factor (BAFF) belonging to the TNF family, CD40L, lymphotoxin β (LTβ), receptor activator nuclear factor ligand (RANKL), or CD30L [178]. The triggering of these cell surface molecules engages the assembly of a signalling complex that involves cellular inhibitor of apoptosis (cIAP1 and cIAP2), TRAF2, and TRAF3 [178]. Both canonical and non-canonical NF-κB activation as well as their dysregulations pathways have been implicated in human haematological malignancies [161,163,178,179].

NF-κB has been found to be constitutively activated in CLL [180,181,182], T-ALL [161,178] and AML [178]. In acute lymphoblastic leukaemia (ALL), the majority of patients present a constitutive activation of the canonical NF-κB pathway in the form of RelA/p50 complexes, which is an important switch to ensure the survival of ALL cells by blocking apoptosis or enhancing cell proliferation [161,178]. Furthermore, kinases acting on IκBα have been found to be activated in most ALL cases [161]. Bcr–Abl expression, as well as increasing the transactivation function of the RelA/p65 subunit of NF-κB, also leads to the activation of NF-κB-dependent transcription causing nuclear translocation of NF-κB in ALL and CML [183,184,185]. NF-κB has also been linked to the expression of multidrug resistance in leukaemia cell lines [186,187]. NF-κB is responsible for the enhancement of drug resistance through the control of the expression of the multidrug resistance gene 1 (mdr1) [187].

The activated form of NF-κB interacts and cooperates with other transcription factors such as STAT3 to modulate specific gene expression [188,189].

The STAT protein family includes seven members that can regulate cancer cell survival, proliferation, and angiogenesis [190]. STAT3 is an essential member of the signal transducer and activator of the transcription (STAT) family of signal responsive transcription factors [188]. STAT 3 remains in an inactive form in the cytoplasm like NF-κB [191,192]. STAT3 is a fundamental signalling intermediate in haematopoietic cells that is activated by recruitment to tyrosine-phosphorylated receptor complexes, including the granulocyte colony-stimulating factor (G-CSF) receptor [193]. STAT3 activation is mediated by the phosphorylation of a crucial tyrosine residue (Tyr 705) that induces STAT3 dimerization through phosphotyrosine-SH2 domain interaction [193,194]. Once dimerized, STAT3 transcription factors enter the nucleus and activate a broad array of target genes. However, unphosphorylated STAT3 is still capable for dimerization and induction of transcription [195,196]. The transcriptional activity of STAT 3 and its DNA binding are further enhanced through serine 727 residues (p-STAT3S727) in leukaemia cells [188,197,198] (Figure 3).

STAT3 has shown a direct link to the development of leukaemia [162], by promoting the proliferation of leukaemia cells, regulating the differentiation and blocking the apoptosis of leukaemia cells [162]. STAT3 is negatively regulated by two types of regulating factors, including the suppressor of cytokine signalling (SOCS) and the protein inhibitor of activated STAT (PIAS), which regulate the active status of STAT3 [199,200]. However, STAT3 can be activated through a number of mechanisms, including through the Ras/mitogen-activated protein kinase (MAPK), Janus kinase (JAK)/STAT3 and non-receptor tyrosine kinase signalling pathways [201]. In cancer cells, a number of cytokines including the IL-6 family, which signal through the gp130 common signalling subunit (IL-6, IL-11, oncostatin M, LIF, CNTF, IL-27 and IL-35), cytokines of the IL-10 family (IL-10, IL-22, IL-19 and IL-20) and growth factors including epidermal growth factor (EGF) family members, hepatocyte growth factor (HGF), VEGF, IL-23 and IL-21 are also capable of activating STAT3 [188,202,203]; moreover, it induces the cell surface growth factor and cytokine receptors, including (EGFR, c-Met, IL-23R) or cytoplasmic proto-oncogenes such as K-Ras, Src and c-Abl, which consequently led to STAT3 phosphorylation [204,205,206,207]. It is also activated by a variety of tyrosine kinase (TK) signalling pathways, such as the Src family kinases (SFKs) and JAK family kinases [201]. This confirms that STAT3 is one of the most commonly activated transcription factors in human cancer [188,191].

Activated nuclear STAT3 has been detected in multiple forms of leukaemia [193,197,208,209,210,211]. The STAT 3 activation has also shown to contribute to multidrug resistance (MDR). The elevation in the activity of STAT 3 enhances leukaemia cells such as AML and CML to be resistant to tyrosine kinase inhibitors (TKI) [212,213]. There are different suggested strategies for STAT3 inhibition, including: firstly, the inhibition of multiple tyrosine kinase pathways of many growth factor receptors such as JAKs, EGFR, and intracellular SFKs which are responsible for the activation/phosphorylation of STAT3 [208]. Secondly, inhibition of protein–protein interactions that involved in STAT3 signalling [208]. Furthermore, inhibition of STAT3-mediated transcription [208]. Finally, inhibition of nuclear import and export (translocation) of STAT3 [208].

NF-κB and STAT3 and their interactions are promising targets for leukaemia treatment [162]. The therapeutic inhibition of either pro-tumorigenic STAT3 or NF-κB signaling is currently being tested in clinical trials, for several cancer types including hepatocellular carcinoma (HCC), colorectal, prostate, breast cancer [156,214] and leukaemia [215,216].

Polyphenols including quercetin and curcumin have also been shown to act indirectly on inhibition of STAT3 in a number of leukaemia cell lines (HL-60, U-937 and K562) [113,125]. Flavopiridol, when used in combination with bortezomib, has also been shown to inhibit STAT3 and STAT5 activity, and induced apoptosis in K562 and LAMA84 leukaemia cell lines [128] (Figure 3).

Quercetin has also been shown to target protein kinases and inhibit protein kinase B (Akt) and extracellular signal-regulated kinases (ERKs) in NB4, HL-60 and THP-1 leukaemia cell lines [114]. Curcumin also inhibits the constitutive activation of pro-survival pathways, some of which are preferentially active in primary B-cell chronic lymphocytic leukaemia (B-CLL) cells, including STAT3, Akt, and NF-κB [63,64]. Resveratrol combined with bestatin downregulates PI3K, Akt and mTOR in K562 and K562/ADR cells [130]. Resveratrol, flavopiridol, and epsilon-viniferin (ε-viniferin) were also shown to induce apoptosis by the reduction of nitric oxide synthases (iNOS) levels in WSU-CLL, ESKOL and B-CLL leukaemia cells [65,66] (Figure 3).

A recent study confirmed that curcumin can affect the apoptosis and invasion of SHI-1 cells in vivo, by the activation of JNK and p38 and the inhibition of ERK and NF-κB signals [140]. Catechin and epicatechin reduce NF-κB activity in PMA-induced Jurkat cells [83]. Flavonoids can modulate NF-κB activation cascade at early phases by affecting IKK activation and the regulation of oxidant levels or at late phases by affecting binding of NF-κB to DNA in Jurkat cells [83]. Quercetin was also found to decrease STAT3 and p-STAT3 at the protein level, resulting in apoptosis in HL60 and U937 cell lines [113]. Apigenin has been shown to target JAK/STAT, inhibit the PI3K/PKB pathway and blocked proliferation through cell-cycle arrest in the G_2_/M phase of the cell cycle and induced caspase-dependent apoptosis in the HL-60 cell line [102] (Figure 3).

Icariside II, an active flavonoid, has been reported to suppress JAK2-dependent STAT3 activation through silencing SHP-1 and inducing apoptosis in U937 [147]. EGCG has been shown to induce apoptosis by targeting JAK2/STAT3/AKT and Bcr/Abl-mediated p38-MAPK/JNK signalling pathways in chronic myeloid leukaemia (CML) cells [217]. Curcumin has also been reported to inhibit anti-apoptotic proteins (e.g., Mcl-1 and XIAP) expression by inhibit STAT in CML [64] (Figure 3).

This suggests that a direct targeting of the pro-inflammatory pathways by polyphenols may have anti-cancer effects in leukaemia.

### 2.2. Polyphenols and Reactive Oxygen Species

The modulation of cellular reactive oxygen species (ROS) and hence cell oxidative stress (OS) is another potential therapeutic target for polyphenols. Oxidative stress is characterised by an imbalance between the production of ROS and a biological system’s ability to neutralize the reactive intermediates to repair oxidative damage [218]. Reactive oxygen species include: hydrogen peroxides (H_2_O_2_); superoxide (O¯_2_); hydroxyl (OH¯) [219]; hydroperoxyl (HOO¯); peroxyl (ROO¯); alkoxyl (RO¯) radicals; and reactive nitrogen species (RNS) such as nitric oxide (NO¯) and peroxynitrite anion (ONOO¯) [15]. Reactive oxygen species production is stimulated by both exogenous factors, such as radiation and therapeutic drugs [218,220]; and endogenous factors such as changes in cellular metabolism [218].

Reactive oxygen species play an important role in physiological and pathophysiological processes, including the regulation of cancer cell proliferation and survival [221]. A moderate increase in ROS production is one of the defining characteristics of cancer cells, including leukaemia cells [222]. This increase in ROS levels results in mitochondrial dysfunction, altered cell metabolism, genetic mutations [223], and an increase in peroxisome activity, cellular receptor signalling, and oncogene activation [224]. It also causes an increase of oxidases, cyclooxygenases, lipoxygenases, and thymidine phosphorylase [224]. This leads to an increase in cell proliferation, a resistance to cell death via apoptosis, and an increase in chemotherapy resistance [218,225].

Conversely, when ROS production is increased dramatically beyond the antioxidant function of cells, oxidative damage can occur, affecting DNA, proteins, and plasma and organelle membranes integrity [219]. The oxidative damage then reduces mitochondrial membrane potential (MMP), resulting in a compromised ATP production and increased cytochrome c release and subsequent apoptosis [226] or cellular senescence [223].

Under normal physiological conditions, the intracellular levels of ROS are carefully regulated, maintaining the internal cellular environment and preventing damage [224]. Levels of ROS are controlled via non-enzymatic molecules, such as glutathione (GSH), vitamins A, C, and E; dietary flavonoids [227]; as well as the induction of phase II detoxifying or antioxidative enzymes such as superoxide dismutase (SOD), catalase, and glutathione peroxidase (GSH-Px) and hemeoxygenase-1 (HO-1), all of which eliminate or inactivate the ROS [227].

**Figure 4:**
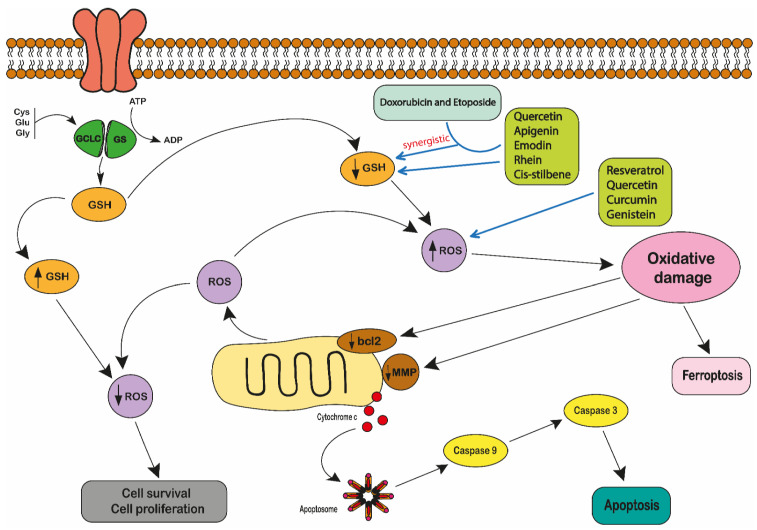
The role of reactive oxygen species (ROS) and glutathione (GSH) in regulation of cell survival and cell death. Glutathione (GSH) (**orange**) is synthesised from glutamate (Glu), cysteine (Cys) and glycine (Gly) through two ATP-dependent steps. They are catalysed by glutamate-cysteine ligase (GCLC) and then glutathione synthase (GS) (**green**). Increased GSH levels (**orange**) lead to reduced ROS (**purple**) production and results in cell survival. In contrast, GSH depletion leads to increases ROS production, redox status alterations, and consequently cell death, whether apoptosis (**blue**) or ferroptosis (**light pink**). Stimulation of reactive oxygen species (ROS) can lead to induction of apoptotic signalling including permeabilisation of the mitochondrial membrane (**brown**), either directly or through Bcl2 (**brown**), which results in the release of cytochrome c (**red**) which in turn activates caspase 9 and 3 (**yellow**), which mediates cell death. Polyphenols (**light green**) alone or in combination with chemotherapy agents (**light blue**) drive an increase in ROS and decrease in GSH levels (**blue lines**) and cancer cell death via apoptosis or ferroptosis.

Of these natural antioxidants, GSH is important in regulating many metabolic and cellular processes and increasing cell survival during periods of exposure to ROS [221,228]. Glutathione (GSH) is synthesised from glutamate (Glu), cysteine (Cys) and glycine (Gly) through two ATP-dependent steps (Figure 4). Firstly, L-glutamate and cysteine are converted to gamma-glutamylcysteine by glutamate-cysteine ligase (GCLC). Then, glycine is added to the C-terminal of gamma-glutamylcysteine in the presence of glutathione synthase (GS). Glutathione protects cells from ROS by reducing the disulphide bonds of the cytoplasmic proteins to cysteines [224]. During this process, GSH is oxidised to form glutathione disulphide (GSSG) in the presence of glutathione peroxidases (GPX) when it catalyses the breakdown of hydrogen peroxide and organic hydroperoxides [224]. The resulting forms of oxidized GSH (GSSG) are then reduced back to GSH by the NADPH-dependent catalysis of the flavoenzyme GSH reductase [229].

Glutathione reductase reduces GSSG back to GSH and refills the GSH pools. Under physiological conditions, GSH almost exclusively exists in its reduced form because of constitutive activity of glutathione reductase in cells [224]. Glutathione S-transferases (GST) are detoxification enzymes that catalyse the conjunction of GSH to a variety of exogenous and endogenous electrophilic compounds [230]. Glutathione S-transferases are overexpressed in a wide variety of tumour types, including leukaemia [231], resulting in increased proliferation via the action of mitogen-activated protein kinase (MAPK) pathways, and its overexpression is associated with increased resistance to chemotherapy [224].

Hence, enhancing the capacity of GSH and its associated enzymes has a fundamental role in protecting normal and cancer cells from redox-related changes or environmental toxins [221]. Therefore, the modulation of cellular GSH has great therapeutic potential in treating cancer. Depleting GSH and GSH-related detoxification pathways is an important strategy to sensitize cancer cells to chemotherapy [221]. Indeed, the depletion of GSH leads to increased ROS production, redox status alterations, and consequently cell death [232]. The type of cell death appears to be linked to the localisation of GSH depletion, with the depletion of cytosolic GSH triggering ferroptosis (Figure 4), whilst the depletion of mitochondrial GSH triggers apoptosis [233]. In contrast, GSH production is increased during oxidative stress and inflammation [232].

Ferroptosis is a form of regulated cell death (RCD) initiated by oxidative perturbations of the intracellular microenvironment that is under constitutive control by GPX4 and can be inhibited by iron chelators and lipophilic antioxidants [234].

Ferroptotic cell death differs from the classic programmed cell death such as apoptosis, autophagy, and necrosis [235]. This form of non-apoptotic cell death is characterised by unique cytological and morphological changes, including a reduction of cell volume and an increase in mitochondrial membrane density [235,236]. Ferroptotic cell death can be induced by iron-dependent lipid peroxide accumulation, caused by the depletion of essential glutathione (GSH) precursors. This GSH depletion can be caused by two classes of small-molecule substances [235]. Class I inhibit system XC^–^ causes a reduction in the intracellular GSH content, causing an oxidation-reduction imbalance in cells through ferroptosis inducers, including erastin, sulfasalazine (SAS), diphenyleneiodonium chloride (DPI2) and buthionine sulfoximine. This causes an oxidation reduction imbalance in cells and cell death [236,237]. Class 2 blocks the GSH-dependent enzyme GPX4 [233,237], by ferroptosis inducers including Ras selective lethal 3 compound (RSL3), diphenyleneiodonium chloride (DPI7, DPI10, DPI12 and DPI13) [233,237] and ultimately leads to an accumulation of toxic lipid ROS (L-ROS) and induces cell death [233,235,236].

Glutathione plays a major role in the removal of ROS from cancer cells, especially leukaemia cells [222]. Many studies have reported that elevated levels of GSH are associated with chemotherapy resistance [221,238,239]. Glutathione binds to or reacts with chemotherapy agents and enhances the depletion of ROS created by the agents, and prevents protein and DNA damage, and can contribute to DNA repair [221].

The modulation of GSH levels has also been shown to influence drug resistant in cancer cells. Drug resistance is frequently associated with the over-expression of P-glycoprotein (P-gp) and/or multidrug resistance proteins (MRPs), which work as cellular pumps, extruding cytotoxic drugs out of tumour cells [232,240]. In contrast, the efflux of GSH, GSSG, and GSH S-conjugates (xenobiotics or metabolites) is achieved via different multidrug resistance proteins [241], with MRP-1, MRP-2 and MRP-4 being responsible for the transport of GSH [232,241,242].

When considering chemotherapy resistance, for example to cisplatin, there are three main mechanisms of action where GSH levels may be involved. Firstly, GSH may serve as a cofactor in facilitating MRP2-mediated efflux in mammalian cells for cisplatin. Secondly, GSH may serve as a redox regulating cryoprotection, based on the observations that many cisplatin-resistant cells overexpress GSH and gamma-glutamylcysteine synthetase (𝛾-GCS). Finally, GSH may function as a copper chelator, which as cisplatin is copper dependant, this will inhibit its actions [220]. For example, an elevated expression of GSTs, combined with high GSH levels, can increase the rate of conjugation and detoxification of chemotherapy agents, thus reducing their effectiveness, and increasing cancer cell survival [221,243].

Likewise, the depletion of GSH levels may also help to sensitize tumour cells to ionizing radiation. This was first demonstrated in CEM and HSB leukaemia cell lines, where it was found that the inhibition of glutathione synthesis, led to a reduction in intracellular GSH levels, and a decreased cell viability, and improved sensitivity to irradiation [244].

GSH levels have been shown to be modulated by polyphenols. Resveratrol has been shown to activate GSH efflux in U-937 cells, resulting in apoptosis [149]. Similarly, chrysin and apigenin have been reported to be the cause of a GSH efflux and to induce a depletion of intracellular GSH levels prostate (PC-3), lung (A549) and myeloid leukaemia (HL-60) cell lines [103] and to lead to an overexpression of Bcl-2-associated X protein (Bax) and apoptosis, without recruiting ROS mechanisms [149,232,245]. This suggests that the depletion of GSH, rather than an increase in ROS, is required for the induction of apoptosis in cancer cells.

The combinations of polyphenols and chemotherapeutic agents have been shown to act synergistically in leukaemia cell lines. Mahbub et al. 2015 showed a synergistic reduction of ATP levels (as a marker of cell viability) and induction of apoptosis when polyphenols (quercetin, apigenin, emodin, rhein and *cis*-stilbene) were combined with topoisomerase II inhibitors (doxorubicin and etoposide) [24] and alkylating agents (cisplatin, cyclophosphamide and chlorambucil) [24]. This was associated with a synergistic depletion in GSH levels and DNA damage in lymphoid (CCRF-CEM and Jurkat) cell lines. Although only quercetin and apigenin showed synergistic actions with these chemotherapy agents in myeloid (THP-1 and KG-1a) cell lines [24,71]. However importantly, when these polyphenols were used alone, there was an increase in DNA damage and an induction of apoptosis in all lymphoid and myeloid leukaemia cell lines tested [23,244]. This was facilitated by a polyphenol-induced decrease in GSH levels [24,67]. Similarly, quercetin has been reported to decrease GSH levels and increase the production of ROS in K562 [129] and MOLT-4 leukaemia cell lines [88]. Resveratrol and genistein also suppressed ROS levels in HL-60, NB4, THP-1, and Jurkat leukaemia cell lines [114,115,116].

However, in contrast, quercetin and curcumin have been shown to reduce, rather than increase ROS levels, in L1210 [97], and HL-60 leukaemia cell lines [108,109]. This dichotomy of action of polyphenols on ROS levels has been reported to be attributed to polyphenol dose within different cell lines [246,247,248,249]. An in vivo mouse model of lung cancer H1299 cell xenografts showed that the effects of epigallocatechin gallate (EGCG) on ROS levels was dose dependent [87]. Low dose treatments caused a protective effect and acted as a free radical scavenger, reducing ROS levels and increasing cell survival; whilst in high EGCG treatments, dose caused an increase in ROS, and a reduction in GSH levels, which led to cell apoptosis [87]. This evaluation included three biochemical parameters: the formation of the oxidative DNA-product, 8-hydroxyl-2′-deoxyguanosine (8-OHdG), which is commonly used as a marker of oxidative stress; the formation of phosphorylated histone 2A variant X (γ-H2AX) which is the cellular marker for the presence of double-strand DNA breaks, that can be caused by ROS; and finally, apoptotic activity, which was measured by the induction of caspase 3 [246]. Hence, there is significant evidence that polyphenols such as EGCG have therapeutic potential in the modulation of ROS and GSH levels, which can ultimately lead to the induction of apoptosis. However, it is important to consider treatment doses, to ensure that the dose is sufficiently high to induce cancer cell death (Figure 4).

### 2.3. Polyphenols and Metabolic Activity

Metabolic activity or more specifically ATP levels are commonly used as an indication of polyphenol treatment on total cell numbers, which is influenced by proliferation rates and viability [250]. A wide variety of polyphenols have been shown to decrease ATP levels in in vitro culture of leukaemia cells [23,24,67,68,69,97,111]. Punicalagin, quercetin, delphinidin, apigenin, emodin, rhein, and *cis*-stilbene have all been shown to decrease cell ATP levels in CCRF-CEM, MOLT-3, Jurkat and HL-60, THP-1, and KG-1a leukaemia cell lines [23,24,68]. Similarly, gallic acid, quercetin and tannic acid reduce ATP levels in L1210 cells [97] and flavonoids derived from thyme have also been shown to decrease ATP levels in CCRF-CEM, THP-1 [69], HL-60 [111] and U-937 [145] leukaemia cell lines.

Polyphenols (quercetin, apigenin, emodin and rhein) also have been shown to synergistically reduce ATP levels (as a marker of cell viability) when combined with alkylating agents (chlorambucil, cisplatin and cyclophosphamide) [71], antimetabolite agents (methotrexate, 6-mercaptopurine and 5-fluorouracil) [23] and topoisomerase II inhibitors (doxorubicin and etoposide) in Jurkat, CCRF-CEM and THP-1 cells [24]. However, in some cell lines, some antagonistic effects were observed when some of these polyphenols were used with some chemotherapy agents, thus care must be taken when selecting the appropriate combination therapies for each leukaemia type [23,24].

It is also important to point out that although polyphenols have been shown to decrease ATP levels in leukaemia cell lines, reduce cell viability and proliferation; they are reported to have limited or no detrimental effect on normal haematopoietic stem cells; suggesting that polyphenols are protective of non-cancerous healthy cells [67,68]. This suggests that metabolic changes seen in cancer cells [251] may be a possible target for polyphenol treatment.

### 2.4. Polyphenols and Cell Cycle Arrest

**Figure 5:**
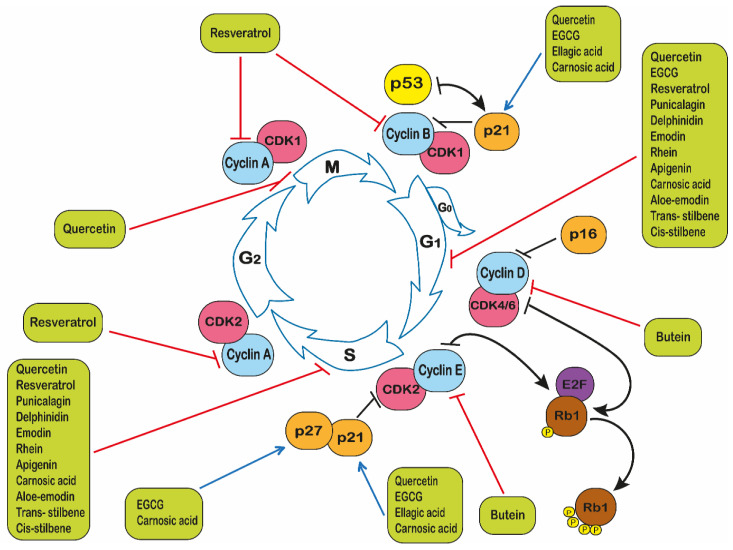
The modulation effect of polyphenols on the cell cycle. Cyclin dependent kinases (CDK) regulate the cell cycle, with cyclins. The CDK inhibitors are regulated by regulatory proteins: p21, p16 and p27 (**shown in orange**). The proto-oncogene P53 (**show in yellow**) regulates transcription factors via induction of p21 which is a CDK inhibitor. The retinoblastoma protein (pRb) (**shown in brown**) is an inhibitor of cell cycle progression from G_1_ to the S phase of the cell cycle by interacting with E2F transcription factors (**purple**), this in turn is regulated by a cell-cycle dependent phosphorylation catalysed by cyclin-dependent kinases in the late G_1_ phase of the cell cycle. The modulation effect of a variety of polyphenols (**lime green**) is shown by the red lines.

Polyphenols have also been shown to impact cell cycle progression. The cell cycle has four basic stages that cells pass through, in order to divide and produce new cells [154] (Figure 5). During this process, cells duplicate their DNA content in S phase, chromosome segregation and cell division occur in M phase, G_1_ is the gap between M phase and S phase, while G_2_ is the gap between S phase and M phase [252]. This process is tightly regulated by a family of serine/threonine protein dependent kinases known as cyclic dependant kinases (CDKs) [154,253]. These enzymes are controlled and activated by the cyclins. The levels of CDKs remain stable, however, their activity levels fluctuate according to the presence of cyclins and inhibitors [254] (Figure 5). CDK inhibitor proteins (CKI) regulate CDK activity via direct binding to CDKs or to CDK-cyclin complexes [253]. There are two families of CKI, the inhibitor of cyclin-dependent kinase 4 (INK4) family and CDK interacting protein/kinase inhibitory protein (Cip/Kip) family [255]. The INK4 family includes p15 (INK4b), p16 (INK4a), p18 (INK4c), and p19 (INK4d) [256], whilst the Cip/Kip family includes p21 (Cip1/Waf1), p27 (Cip2), and p57 (Kip2). These CKIs can inhibit the activity of CDK-cyclin complexes and stop the progression of the cell cycle and the production of new cells by mitosis [257,258] (Figure 5). The cell cycle is regulated by a number of key proteins and enzymes which regulate transfer between each phase. The proto-oncogene p53 regulates transcription factors via the induction of p21. The retinoblastoma protein (Rb) is an inhibitor of cell cycle progression from G_1_ to the S phase of the cell cycle by sequestering E2F transcription factors; this in turn is regulated by a cell-cycle dependent phosphorylation catalysed by cyclin-dependent kinases in the late G_1_ phase of the cell cycle [259] (Figure 5).

Emodin, quercetin, apigenin, rhein, aloe-emodin, trans- and *cis*-stilbene have all been shown to cause cell cycle arrest in G_0_/G_1_ phase of the cell cycle in CCRF-CEM, THP-1 and KG-1a leukaemia cell lines; and S phase arrest in Jurkat leukaemia cells [24,67]. Similarly, in 232B4 cells, quercetin caused G_0_/G_1_ cell cycle arrest [61]. Treatment of polyphenols from pomegranates: punicalagin, quercetin and delphinidin also showed G_0_/G_1_ and S phase cell cycle arrest in Jurkat, MOLT-3, HL-60, THP-1 and KG-1a leukaemia cell lines [68,260,261] (Figure 5). Likewise, treatment with quercetin has also been shown to cause cell cycle arrest in G_2_/M phase in both MOLT-4 [88,89] and L1210 leukaemia cells [98]. Similarly, resveratrol has also been shown to cause G_0_/G_1_ cell cycle arrest in HL-60, 232B4 and WIL2-NS leukaemia cells [61,117,153]; whilst S phase arrest was seen in KG-1a cells [135] (Figure 5).

At a molecular level, studies have reported the ability of polyphenols such as resveratrol to arrest cell cycle progression in HL-60 leukaemia cells by inducing the overexpression of cyclins A and E, resulting in the accumulation of the cells in the G_1_/S phases [118]. Flavopiridol has shown its ability to inhibit CDKs including CDK1, CDK2, CDK4, CDK6, and CDK7 in different solid tumour cell lines [118,262,263,264].

Quercetin in combination with ellagic acid has been shown to increase p21Cip1/Waf1 and MAP kinases in MOLT-4 cells [88], whilst resveratrol has been shown to enhance cell cycle arrest by inhibiting cyclins A and B in Jurkat, U-937, K562, WSU-CLL, HL-60, THP-1 and KCL22 cells [96].

Similarly, epigallocatechin gallate has been shown to increase the expression of p21 in NB4 and HL-60 cells [110], and p27Kip1 in both Jurkat [84] and Raji cells [144]. Meanwhile, carnosic acid induced G_1_ phase cell cycle arrest, which was associated with increased p21Cip/1Waf1 and p27Kip1 in HL-60 and U-937 cells [105]. CDK4 and CDK6 and their regulatory proteins (cyclin D1, cyclin D2 and cyclin E) expression levels have been reported to be downregulated by the polyphenol butein in MT-4 and TL-Oml cells [92]. This suggests that polyphenols are capable of both reducing CDKs, whilst increasing the production of CKIs, resulting in cell cycle arrest, highlighting the therapeutic potential of polyphenols in preventing cell cycle progression and cell division in leukaemia.

### 2.5. The Pro-Apoptotic Effect of Polyphenols in Leukaemia

**Figure 6:**
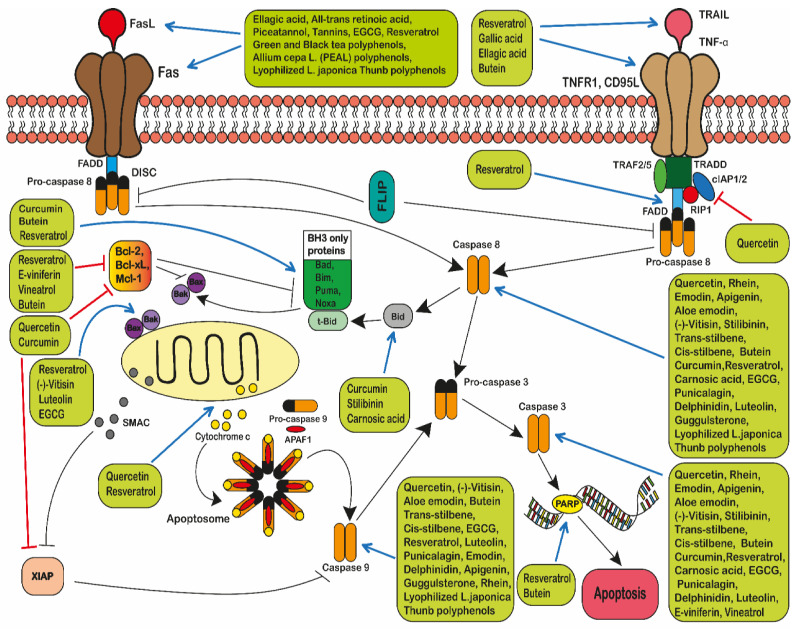
The modulation effect of polyphenols on the intrinsic and extrinsic pathways of apoptosis. The extrinsic death receptor pathway activates by the death receptor ligands such as FasL (**dark red**), TNF-α or TRAIL (**light red**). The binding of FasL to Fas (**dark brown**) recruits FADD (**light blue**) and pro-caspase-8 (**orange and black**), forming the DISC complex, which activates caspase-8 and then caspase 3. The binding of TNF-α (**light red**) to TNFR1 (**light brown**) recruits TRADD (**dark green**), RIP (**red**), TRAF2/5 (**light green**) and cIAP1/2 (**blue**) to form the complex, which in turn binds to pro-caspase 8 and then activates caspase 8. The intrinsic death receptor pathway is initiated by BH3-only protein (**green**), which can inactivate Bcl-2 (**dark orange**) and prevent Bcl-2 from effectively neutralizing Bax (**dark purple**) and Bak (**light purple**) and activates them. The Bax and Bak activation on the mitochondrial membrane leads to release of second mitochondria-derived activator of caspase a mitochondrial protein (SMAC) (**dark grey**) and cytochrome c (**yellow**) to the cytoplasm. The cytoplasmic cytochrome c then associates with Apaf-1 (**red**) and pro-caspase 9 to form the apoptosome, which activates caspase 9 and caspase 3, leading to apoptosis. Caspase 3 in turn initiates the cleavage of the nuclear enzyme PARP (**yellow**) which recognises damaged DNA, and initiates apoptosis if DNA damage is not repaired. Finally, SMAC is able to regulate apoptosis by inhibiting the inhibitor of apoptosis protein (XIAP). The effect of a variety of polyphenols (**shown in lime green**) is shown on both genes and proteins level regulating the intrinsic and extrinsic pathways of apoptosis. The blue arrows indicate the induction or activation of a protein or genes; whilst the red line shows the depletion or inhibition of a protein or gene expression.

There are two main types of apoptosis: type I or the extrinsic apoptotic pathway, which is death-receptor-mediated and dependent on caspase 8 activation; and the type II or the intrinsic apoptotic pathway which is triggered by intracellular signals (e.g., DNA damage) and is caspase 9-dependent and mitochondria-mediated [265,266] (Figure 6).

#### 2.5.1. The Extrinsic Pathway

The Extrinsic Pathway is defined as a “specific variant of regulated cell death initiated by perturbations of the extracellular microenvironment detected by plasma membrane receptors, propagated by caspase 8 and accelerated via executioner caspases including caspase 3” [234].

Extracellular signals mediate apoptosis via the extrinsic pathway. Death receptors (DRs) are members of a subset of the tumour necrosis factor (TNF) receptor superfamily [265]. The extrinsic pathway is initiated via the binding of death ligands to their respective death receptors, such as FasL, tumour necrosis factor-alpha (TNF-α) [267], lymphotoxin-alpha (LT-α), TNF-like protein-1A (TL1A), and Apo2L/TNF-related apoptosis-inducing ligand (TRAIL) [268]. Adaptor proteins are then recruited to the Fas-associated death domain (FADD) and TNF receptor-associated death domain (TRADD) on the death receptor [269]. During the extrinsic pathway, procaspase 8 and 10 initiate and bind to the adaptor proteins to form the death-inducing signalling complex (DISC) which in turn causes their activation [269]. The formation and mechanism of DISC is regulated by the cellular FADD-like IL-1β-converting enzyme-inhibitory protein (c-FLIP) [265]. Next, caspase 3, 6 and 7 are activated together with a cascade of apoptosis proteins which degrade the cell, leading to cell death [265,270]. This causes direct activation of the executioner caspase 3, or alternatively, it may activate caspase 9 and activation of the intrinsic apoptosis cascade.

Polyphenols extracted from *lyophilized Lonicera japonica* (PELJ) and ellagic acid, together with all-trans retinoic acid, have been shown to trigger FasL signalling-dependent apoptosis in Jurkat, U-937 and HL-60 cells [122,150]. Resveratrol has been shown to enhance FasL expression, and Fas signalling and the activation of caspase 8 in HL-60, U-937 and Jurkat cells [75,115,271]. Resveratrol was also shown to cause S-phase cell cycle arrest, prior to Fas independent apoptosis in CCRF-CEM-C7H2 cells [74]. Similarly, Reis-Sobreiro et al. 2009 showed that the apoptosis induced by resveratrol in leukaemia cell lines (Jurkat, MM1S, MM144 and U-266) was associated with the activation of the Fas/CD95 death receptor [86]. However, in contrast Wang et al. 2005 found that apoptosis triggered by resveratrol in Jurkat cells, was associated with a FADD protein-dependent mechanism, without any involvement of CD95L, TNFα and TRAIL death receptors [85]. Resveratrol was also shown to increase the mRNA expression of TRAIL receptors in KG1-a cells [134] and induce Fas-mediated apoptosis in SEM, CEM, Nalm-6, REH, RS4;11 and MV4;11 [75], K562 and HSB-2 leukaemia cell lines [81]. Resveratrol also induced apoptosis in K562 and Adriamycin-resistant K562/ADR cells by triggering caspase 8 [130]. Moreover, (-)-Vitisin B, which is a resveratrol tetramer, was also shown to induce caspase 8 activity in HL-60 cells [120].

Other polyphenols have also been shown to induce apoptosis via the Fas-FasL system in leukaemia cells. Curcumin and carnosic acid have been shown to activate caspase 8 in KG-1a, HL-60, U-937, NB-4, murine C1498 and TIB-49 cells, and in vivo in a systemic AML model and peritoneal AML tumour model [106]. Likewise, curcumin, silibinin and carnosic acid have been reported to activate caspase 8 in HL-60 and KG-1a cells [107]. EGCG has also been shown to induce apoptosis by activating the Fas-associated receptor and caspase 8 in vitro in K562 [126,127], NB4, and HL-60 cells [110] and in vivo in a non-obese diabetic/severe combined immunodeficiency (NOD/SCID) mice model [272]. Gallic acid and ellagic acid alone [273] and in combination with all-trans retinoic acid [122] have been shown to trigger death receptor-induced apoptosis in HL-60 leukaemia cell lines. Likewise, butein-upregulated DR5 mRNA expression, induced TRAIL-mediated cell death and caspase 8 activation in K562, U-937, THP-1, HL-60 and Jurkat cells [82,104].

Piceatannol [148], green and black tea [274], woodfordin I extract (high in tannins) [123], and *Allium cepa L*. (PEAL) polyphenols [141] also increase Fas/FasL and induce apoptosis in K562, U-937, THP-1 and HL-60 cells. Meanwhile, quercetin [24,124], emodin, apigenin, rhein, aloe emodin and *cis*-stilbene [24] guggulsterone [146] and polyphenols that were extracted from *lyophilized Lonicera japonica* (PELJ) [150] induce both caspase 8- and 9-mediated apoptosis in U937, Jurkat, CCRF-CEM, THP-1, KG-1a cells.

These studies provide considerable evidence that polyphenols can induce extrinsic apoptosis, through the activation of death receptors, upregulation of pro-apoptotic proteins and induction of caspase 8 and 9.

#### 2.5.2. The Intrinsic Pathway

The intrinsic pathway is defined as a “type of regulated cell death that is initiated by perturbations of whether the extracellular or intracellular microenvironment, determined by MOMP, and accelerated via executioner caspases including caspase 9 and 3” [234].

Intrinsic apoptosis is stimulated following internal cell signals such as DNA damage [269,275] by apoptosis inducing factor (AIF) and endonuclease G (ENDOG) relocation to the nucleus, where they mediate large-scale DNA fragmentation known as caspase-independent apoptosis [276]. Alternatively, caspase-dependent intrinsic apoptosis is initiated via the recruitment of caspases, Bcl-2 family of proteins and p53 [276,277]. B-cell lymphoma-2 (Bcl-2) protein family is one of the most important protein families responsible for the regulation of the intrinsic pathway [265]. Apoptotic stimuli result in the upregulation of the pro-apoptotic BH3-only proteins, which then inhibit the anti-apoptotic Bcl-2 proteins and reactivate both pro-apoptotic Bax and Bak proteins [269]. Bax is regulated by the tumour suppressor gene p53 [278]. Once Bax and Bak are activated, they result in mitochondrial outer membrane permeabilization (MOMP), which is the defining event of caspase-dependent intrinsic apoptosis and is considered as the point of no return [279]. The mitochondrial permeabilization leads to the release of three intermembrane proteins: cytochrome c, second mitochondria-derived activator of caspase (SMAC) and mitochondrial serine protease (Omi). As soon as cytochrome c is released, it binds to apoptotic protease-activating factor-1 (APAF-1) and dATP, to form an apoptosome. Now in the presence of the apoptosome, procaspase-9 is converted into caspase 9 [265,280]. Caspase 9 in turn activates caspases 3 and 7 [281]. These executioner caspases begin to break down proteins leading to cell death [279] (Figure 6).

Intrinsic apoptosis could include additional steps to confirm cell death. SMAC and Omi are activated during apoptosis to inhibit inhibitor of apoptosis proteins (IAP), so that apoptosis proceeds once the apoptosome is formed and X-linked inhibitor of apoptosis protein (XIAP), which is an endogenous inhibitor of caspase function [265]. Although the activation of caspases is important during the apoptosis process, most cells can undergo apoptosis by mitochondrial outer membrane permeabilization without the activation of caspases. Once the permeabilization of the mitochondrial membrane has occurred, it loses function, leading to cell death [279]. The permeabilization of the mitochondrial membrane can also be induced by the accumulation of calcium ions, deprivation of growth factors, DNA damage or oxidants, and microtubule targeting drugs [280]. However, some cells, particularly cancer cells and neurons, have the ability to survive mitochondrial outer membrane permeabilization [279] (Figure 6).

A number of polyphenols have been shown to induce intrinsic apoptosis in leukaemia cells. Quercetin has been shown to increase the release of cytochrome c in K562 and U-937 cells [124,129] and decrease Bcl-2, Bcl-xL, XIAP, cIAP-1 and cIAP-2 levels and activate caspase 8 and 3 in U-937 cells [124] and caspase 8, 9 and 3 in CCRF-CEM, Jurkat, KG-1a, THP-1, MOLT-3 and HL-60 cells [24,67,68]. Quercetin has also been reported to enhance apoptosis through the downregulation of Mcl-1 expression in B cells isolated from CLL patients [19]. Similarly, curcumin has been shown to promote pro-apoptotic activity through the inhibition of XIAP and Mcl-1, and increase Bim levels in BKS-2 and WEHI-231 cells [152]. Likewise, when curcumin was combined with silibinin and carnosic acid, levels of caspase 8, 9, 3 and Bid were increased in HL-60 and KG-1a cells [107]. Resveratrol has also been shown to activate caspase 8, 9 and 3, as well as increase Bax production and the release of cytochrome c in K562, HSB-2 [81], WSU-CLL and B-CLL cell lines [66]. These increases in caspase activity have also been shown to be accompanied by the cleavage of poly(ADP-ribose) polymerase (PARP), and cause growth arrest and DNA damage (measured as GADD45 expression) in HL-60, K562, OCI/AML3, MOLT-4, Jurkat, SEM, RS4:11, MV4:11, REH, NALM-6 and CEM cells [76]. Furthermore, resveratrol has been shown to increase the production of pro-apoptotic proteins Bax, Bad and Bim in MOLT-4, Jurkat, CEM-C-15 and CEM-C7-14 cells [77], and downregulate Bcl-2 expression in L1210 [99], KG-1a and HL-60 [119,135], Jurkat, Kasumi-1 and SUP-B15 [87], WSU-CLL, B-CLL and ESKOL cells [66].

(-)-Vitisin [120], luteolin [112] and epigallocatechin gallate (EGCG) [110] have also been shown to induce apoptosis via the activation of caspase 8, 9 and 3 and increase Bax in HL-60 cells. A combination of polyphenols, e-viniferin and vineatrol also downregulated Bcl-2 expression and activated caspase 3 in ESKOL and WSU-CLL cells [80]. Similarly, punicalagin and delphinidin activated caspase 8, 9 and 3 and induced apoptosis in CCRF-CEM, MOLT-3, HL-60 and THP-1 cells [68]. Meanwhile, emodin, apigenin, rhein, aloe-emodin, *trans*- and *cis*-stilbene also activated caspase 3, 8 and 9 in CCRF-CEM, Jurkat, THP-1 and KG-1a cells [24,67]. Finally, butein has also been shown to downregulate Bcl-2; increase Bad, Bid, and PARP cleavage; and activate caspase 8, 9 and 3 in K562, THP-1, HL-60 and U-937 cells [104].

These studies demonstrate that polyphenols can induce intrinsic apoptosis, via the upregulation of pro-apoptotic and the downregulation of anti-apoptotic proteins causing a decrease in mitochondrial membrane integrity and induction of caspases 9, 7 and 3.

#### 2.5.3. Autophagy

Autophagy is a form of regulated cell death that depends on the autophagic machinery or components thereof in its mechanisms [234].

**Figure 7:**
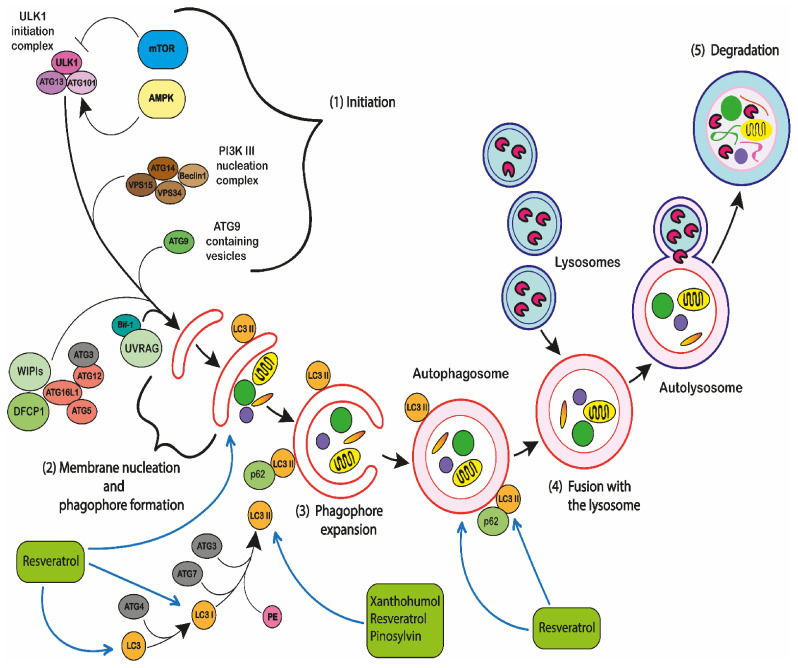
Stages and process of the autophagy. The process of the autophagy includes five stages (**1**) Initiation, (**2**) membrane nucleation and phagophore formation, (**3**) expansion of phagophore, (**4**) fusion with the lysosome, and (**5**) degradation. The autophagy initiation is mainly regulated by the mammalian target of rapamycin (mTOR) complex 1 mTOR (**blue**) as an inhibitor and AMP-activated kinase (AMPK) (**light yellow**) as an activator. The inhibition of mTOR and/or activation of AMPK leads to activation of the Unc-51-like kinase 1 (ULK1) complex (**Purple**), which includes ULK1, autophagy-related protein 13 (ATG13), and ATG101. This leads to triggering of the autophagy machinery and enables nucleation of the phagophore and regulated by three regulatory protein complexes. The class III PI3K (PI3KC3) complex (**brown**) involves vacuolar protein sorting 34 (VPS34), (VPS15), Beclin 1 and ATG14. The autophagosome-specific phosphatidyl-inositol-3-phosphate (PI3P)-binding complex (**green**) includes WD-repeat domain phosphoinositide-interacting proteins (WIPIs) and double FYVE domain-containing protein 1 (DFCP1). WIPIs then bind to ATG12-ATG5-ATG16L1 complex (**light red**), that in turn enhances the LC3 conjugation (**orange**). LC3 is converted into LC3-I by ATG4 (**grey**) and then conjugated with phosphatidylethanolamine (PE) (**pink**) and in the presence of ATG3 and ATG7 (**grey**) to form LC3-II, which is the characteristic signature of autophagosomal membranes. Different cellular membranes involve the elongation of the autophagosomal membrane such as the lipid bilayers, which are delivered by ATG9-containing vesicles (**dark green**). Now, the membrane of the autophagosomal seals around the structure are to be removed from the cell. The autophagosome fuses with the lysosome which destroys the contents. Finally, the autophagic cargo is degraded.

There is also evidence that polyphenols can induce autophagy. Autophagy is described as the molecular survival mechanism in which cells autodigest cellular components or organelles in order to survive periods of stress or starvation. These components can be recycled to aid cell survival [282,283]. However, if the stress levels continue, this will cause cells to progress from autophagy to apoptosis and cell death (Figure 7). Therefore, the modulation of autophagy is a potential target in cancer therapeutics.

Autophagy involves two main intracellular processes; firstly, there is the formation of an autophagosome. This is a double-membrane vesicle which surrounds the cellular structure or protein which is to be degraded [284]. Next is the production of lysosomes which fuse to the autophagosome and cause the degradation of the encapsulated structure [284]. Autophagy is a highly regulated process, involving many cellular functions, including the regulation of cell growth and survival [285]. Malfunctions in these processes can result in many pathophysiology changes, including cancer [284]. Therefore, autophagy is activated by tumour suppressor genes, and downregulated by oncogenes [286].

Autophagy is controlled by a group of autophagy-related genes (Atg genes) that are required for autophagosome formation [287,288,289]. There are also a number of proteins that play a central role in the regulation of initiation of autophagy; these include: mammalian target of rapamycin kinase (mTOR) which acts as a sensor for growth factors and nutrient availability and suppression of mTOR during starvation or cell stress will activate autophagy [290]. Similarly, AMP-activated protein kinase (AMPK) is a key energy sensor and regulates cellular metabolism to maintain energy homeostasis. An increase in AMPK can also induce autophagy [290].

Beclin 1 interacts with its target lipid kinase class III phosphatidylinositol 3-kinase (PIK3C3)/VPS34 and regulates its activity, thus it works as an essential regulator of autophagy downstream of mammalian target of rapamycin (mTOR) [287,291]. Beclin 1 is also important for the recruitment of autophagy proteins during the formation of autophagosome [287]. The Beclin 1-Vps34/PI3KC3 interaction is promoted by its activating molecule beclin1-regulated autophagy (Ambra1), which is another essential regulator during the process of autophagy [292,293].

The induction of autophagy occurs through mTOR inhibition or AMPK activation [290]. Autophagy is initiated by the Unc-51 like autophagy activating kinase 1 (ULK1) (human homolog of ATG1) complex. This is formed by the combination of ULK1, autophagy-related protein 13 (Atg13), and 17 (Atg17), and initiated by stress signals from the mammalian target of rapamycin (mTOR) complex 1 (mTORC1) [294] (Figure 7). Due to stress or nutrient deprivation, mTORC1 is inhibited, increasing ULK1 activity, and this leads to the initiation of autophagosome formation [295,296]. This is started with the creation of a vacuolar sorting protein 34 (Vps34), class III phosphoinositide 3-kinase (PI3K) and beclin 1 complex [297]. Now, during the initiation phase of phagosome formation, a complex of the Atg5-Atg12-Atg16 proteins is formed, which contributes to the conversion of cytosolic-associated protein light chain 3 (LC3-I) to its lipidated membrane-bound form LC3-II [298] (Figure 7). Then, LC3-II is attached to phosphatidylethanolamine (PE) and incorporated into the membrane by an Atg7- and Atg3-dependent activation and transfer cascade that leads to cleavage of LC3 by the cysteine protease Atg4 [299]. LC3 in turn combines with phosphatidylethanolamine and integrates into the membrane [299]. LC3-II remains attached to the surface of mature autophagosomes until the formation of autolysosomes is completed, which monitors autophagy [294] (Figure 7). After the outer autophagosomal membrane fuses with lysosomes, the inner autophagic body membrane and the cargo are degraded by a putative lipase and Atg15 and resident hydrolases respectively (Figure 7) [300]. The macromolecules derived from the cellular components are now recycled back into the cytoplasm by numerous permeases, including Atg22 (Figure 7) [300,301]. Alternatively, autophagy has been found to be promoted by AMPK through mTOR-dependent transcription factor EB (TFEB) activation and increasing the co-activator-associated arginine methyltransferase 1 (CARM1) levels, which is an important cofactor for TFEB transcription [295].

Other proteins that play a role in modulating autophagy include p53, which activates numerous autophagy inducers, including DNA damage-regulated autophagy modulator 1 (DRAM1), Sestrin 2 [286], and the unfolded protein response, which is a component of the endoplasmic reticulum (ER) stress pathway [294]. RAS membrane-anchored protein [286], Sequestosome 1 (SQSTM1)/p62 can assist mTORC1 activation on lysosomes, enables possesses LC3-interacting region and can also act as a cargo receptor for the autophagy of ubiquitinated proteins [302]. Following the binding to the ubiquitinated cargos, p62 undergoes oligomerization and delivers the cargo aggregates to the autophagosome via interacting with the autophagosomal membrane protein LC3 [303]. There is also evidence that the increases in p62 protein levels associates with induction of autophagy [90]. There are several preclinical studies which have shown that the inhibition of autophagy improved the chemosensitivity and enhanced tumour cell death [294].

#### 2.5.4. Autophagic Cell Death

Autophagy is also believed to drive apoptosis [304,305,306,307,308,309]. This is described as autophagic cell death (ACD) [305]. During ACD, cells undergo apoptosis either by inhibition, depletion or deletion of autophagy genes and proteins [305,308]. For example, during autophagy, the antioxidant catalase can be reduced, resulting in an increase of ROS driven cell death, removing pro-apoptotic proteins such as Bax and Bak or the increase in caspase inhibitors [305,307,310,311,312]. Alternatively, apoptosis can be induced by the non-selective degradation of cellular components, such as mitochondria; to such a level that the cell can no longer survive [305,307,310,311,312].

One situation where autophagy may cause cell death is where cells no longer have the ability to activate intrinsic apoptosis, which is usually the preferred mechanism of death [306]. However, this scenario does not mean that autophagy is directly responsible for activating apoptosis; the process might be indirect [307]. For example, the autophagic removal of essential cellular components such as mitochondria will ultimately result in cell death [309]. Moreover, autophagy may contribute to an increase in ATP supplies, which are required for membrane blebbing during apoptosis [304]. Interestingly, both autophagy and apoptosis respond to similar stresses [313] and there is considerable evidence that autophagy, drives apoptosis under certain conditions [305,306]. Autophagy is likely to occur at early time points and during low levels of stress; whilst apoptosis occurs at late time points when stress levels are high [305,314].

**Figure 8:**
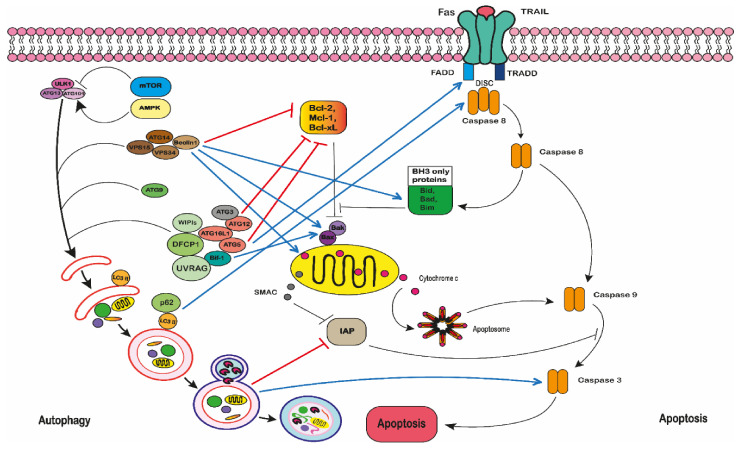
Autophagy mediates programmed cell death. The extensive proteins and pathways crosstalk between autophagy and apoptosis, which include the Beclin 1-BCL-2 interaction; caspase-mediated Beclin 1 cleavage; ATG12- ATG3 conjugation; ATG12-Mcl-1 interaction; ATG5-FADD interaction. (**red lines**) represent inhibitory interactions, (**while blue lines**) with arrows represent stimulatory interactions.

Multiple direct and indirect interactions have been described, suggesting that there are mechanistic interactions between apoptosis and autophagy [307] and this can lead to apoptotic cell death [287,315] (Figure 8).

Firstly, autophagy machinery regulates apoptotic proteins. For example, a number of conjugates can be formed between autophagy and apoptotic proteins. These include: ATG3-ATG12-Bcl-xL, beclin 1-Bcl-2, beclin 1-Bim, beclin 1-Bak/Bax, beclin 1-mitochondria; beclin 1-cytochrome c; Bif1-Bak/Bax; ATG12-Bcl-2; ATG12-Mcl-1, ATG5-FADD [287,304,305,307,313,314,316,317]. This demonstrates how these two processes are interlinked and how ACD can be regulated (Figure 8).

Alternatively, ACD can be induced by the activation of caspase 8. This can occur by the formation of the death inducing signalling complex (DISC-like complex) on the autophagosomal membrane, through an interaction of the adaptor protein FADD and ATG5 [287]; or by the formation of an ATG5, LC3 and p62 platform; these subsequently lead to caspase 8 activation and extrinsic apoptosis [315]. Similarly, the inactivation of beclin-1 can cause an increase in caspase 9 and 3, and intrinsic apoptotic cell death [304,318,319] (Figure 8).

Apoptosis can also be induced by the autophagic removal of apoptotic proteins within the autophagosome. For example, the degradation inhibitor of apoptotic proteins (IAP) ultimately leads to apoptosis [287,315] (Figure 8). Plus, as soon as apoptosis is initiated, this will cause cytoprotective molecules to become cytotoxic, which prevents cytoprotective mechanisms such as autophagy [305].

Secondly, apoptosis machinery can be regulated by autophagy proteins. Autophagy proteins can mutate p53 and directly regulate Bcl-2 family proteins, PI3K, AKT, and FLIP [287,304,305,308,310,313,320]. Similarly, numerous autophagic proteins have been identified as targets for caspase-mediated cleavage. Those autophagy proteins targeted for caspase cleavage include: beclin 1, VPS34, ATG3, ATG4D and AMBRA1 [287,289,292,305,314,321,322].

Autophagy has also been shown to be a target for polyphenols in leukaemia cells. Resveratrol has been shown to increase in LC3-II and p62 protein expression and induced autophagy in MOLT-4 and HL-60 cells [90] (Figure 7). Resveratrol also induced autophagy in imatinib-sensitive (IM-S) and resistant (IM-R) chronic myelogenous leukaemia (K562) cells via JNK-mediated p62/SQSTM1 expression and AMPK activation [133] (Figure 7). Similarly, resveratrol increased the formation of autophagosomes and phagophore and LC3 production in K562, HL-60, OCI/AML3, OCIM2, MOLT-4, CEM, Jurkat, SEM, RS4:11, MV4:11, REH and NALM-6 cell lines [76] (Figure 7). Resveratrol and pinosylvin also showed enhancement of LC3-II and LC3 accumulation in U-937 and THP-1 cells [142]; and an increased ratio of LC3-II: LC3-I in MOLT-4, Jurkat, CEM-C1-15 and CEM-C7-14 cells [77]. Xanthohumol also led to LC3-II accumulation in HL-60 cells [121] (Figure 7). This demonstrates that polyphenols have the potential to induce autophagy, and drive autophagic cell death.

### 2.6. Interaction of Chemotherapy and Multidrug Resistance

One major problem with the use of chemotherapy treatments is the emergence of multidrug resistance (MDR) [323]. MDR affects drug uptake, drug efflux, activation of DNA repair mechanisms, activation of detoxifying systems and the evasion of drug-induced apoptosis [324]. In leukaemia, drug influx and efflux mechanisms decrease the intracellular concentration of chemotherapy agents [323,325]. This is mediated by ATP-binding cassette (ABC) transport proteins, which includes: ABCB1 (known also as P-Glycoprotein [P-gp] or multidrug resistance protein MDR1); ABCC1 (known also as MDR-associated protein [MRP] 1), and lung resistance protein (LRP) [325]. The ABCB1 protein is located in the plasma membrane [326], and decreases the intracellular concentration of drugs [327]. The expression of these proteins is associated with the obstruction of a wide range of hydrophobic anti-cancer drugs [328]. The ABCC1 protein is also a drug-efflux pump [329], which is ATP-dependant [330]. Meanwhile, LRPs are nuclear-cytoplasm transport proteins [327] and their expression is associated with resistance to chemotherapy agents, including doxorubicin, vincristine and platinum-based chemotherapy compounds such as cisplatin and carboplatin [331].

There is considerable evidence to suggest that polyphenols could prevent and reverse MDR. Resveratrol has been shown to inhibit the expression of MDR1 (or P-gp) in CCRF-CEM cells [70], arabinoside antimetabolite (Ara-C)-resistant AML-2/WT cells and doxorubicin resistant acute myeloid leukaemia cell lines (AML-2/DX30, AML-2/DX100 and AML-2/DX300) [100]. Furthermore, the combination of resveratrol and bestatin reduces MDR1 function and expression at mRNA and protein levels in K562 and K562/ADR cells [130]. EGCG, tannic acid and curcumin have also been shown to inhibit the activity of MDR1 proteins when combined with doxorubicin in CCRF-CEM cells [24]. Furthermore, EGCG alone has been reported to inhibit both MDR proteins: ABCB1 and ABCC1 in NB4 and HL-60 cells [110]. These findings suggest that polyphenols may help prevent and possibly reverse multidrug resistance.

## 3. Discussion

Polyphenols provide a broad spectrum of biological activity which could be utilised in the treatment of cancer. The pharmacokinetic, pharmacodynamic and safety properties of polyphenols are currently being investigated in clinical trials on different diseases and are still unknown [271,332,333,334]. However, based on limited biological data in humans, some polyphenols are considered pharmacologically safe [271], although little is known about the bioavailability or metabolic forms in which polyphenols are found in blood and tissues. In addition, several in vitro studies confirm their chemopreventive potential, by showing their antioxidant and anti-inflammatory activity. Furthermore, there is evidence that polyphenols are pro-apoptotic and pro-autophagic and sensitize cancer cells to anti-cancer drugs, enhancing their action [23,24].

Leukaemia might benefit from a treatment or pre-treatment with polyphenols during a combined therapy with standard chemotherapy agents. The pro-apoptotic and pro-oxidant effects represent a useful tool to sensitize cancer cells to a subsequent chemotherapy as well as the enhancement of different molecular pathways [76]. Polyphenols also show potential in protecting or possibly reversing multidrug resistance [335]. However, current evidence is predominately from in vitro studies on leukaemia cell lines and thus in vivo studies and clinical trials are needed to investigate the potential of these polyphenols.

## 4. Conclusions

The pharmacological properties of polyphenols, especially anti-tumour activity, antioxidant, and anti-inflammatory activities, support the use of these agents as complementary nutritional/pharmacological biomolecules. However, more attention should be paid to the bioavailability and toxicity in humans, as they are the key to developing treatments [335]. Given the fact that polyphenols regulate multiple molecular targets and signalling pathways, it is important to further elucidate these mechanisms to determine potential interactions with chemotherapy agents. In keeping with the evidence presented here, polyphenols can be considered as a therapeutic approach for the treatment of leukaemia alone and in combination with chemotherapy agents, although antagonistic actions must also be investigated [23,24,336].

## Figures and Tables

**Table 1:** Summary of leukaemia cells lines discussed within this review, including the species of origin, designated abbreviation, commercial source or reference where the cell line was introduced and specific polyphenols that affect the cell lines.

CANCER TYPE	CELL LINE	DESCRIPTION	SPECIES	SOURCE OR REF	POLYPHENOLS
**LYMPHOID** **LEUKAEMIA**	232B4	Chronic lymphocytic leukaemia	Human	Wendel-Hansen et al. 1994 [60]	Quercetin [61] Resveratrol [61]
	B-CLL	Chronic lymphocytic leukaemia	Human	Hoogendoorn et al. 2004 [62]	Curcumin [63,64] Epsilon-viniferin (ε-viniferin) [65] Flavopiridol [65] Quercetin [19] Resveratrol [66]
	CCRF-CEM	Acute lymphocytic leukaemia	Human	ATCC	Aloe-emodin [67] Apigenin [67] Delphinidin [68] Emodin [67] Flavonoids [69] Punicalagin [68] Quercetin [67,68] Rhein [67] Resveratrol [70] Trans-Stilbene [67] *cis*-Stilbene [67] Quercetin, apigenin, emodin, rhein or *cis*-stilbene plus doxorubicin or etoposide [24] Quercetin, apigenin, emodin, rhein, or *cis*-Stilbene plus chlorambucil, cisplatin or cyclophosphamide [71] Quercetin, apigenin, emodin, rhein, or *cis*-stilbene plus methotrexate, 6-mercaptopurine or 5-fluorouracil [23] EGCG, tannic acid or curcumin plus doxorubicin [72]
	CCRF-CEM-C7H2	Acute lymphoblastic leukaemia	Human	Strasser-Wozak et al. 1995 [73]	Resveratrol [74]
	CEM	Acute lymphoblastic leukaemia	Human	ATCC	Resveratrol [75,76]
	CEM-C1-15	Acute lymphoblastic leukaemia	Human	ATCC	Pinosylvin [77] Resveratrol [77]
	CEM-C7-14	Acute lymphocytic leukaemia	Human	Gu et al. 2019 [78]	Pinosylvin [77] Resveratrol [77]
	ESKOL	B-lymphoblastoid hairy cell leukaemia cell lines	Human	Harvey et al. 1991 [79]	Resveratrol [66] e-Viniferin plus Vineatrol [80]
	HSB-2	Acute lymphoblastic leukaemia	Human	ATCC	Resveratrol [81]
	Jurkat	Acute lymphocytic leukaemia	Human	ATCC	Aloe-emodin [67] Apigenin [67] Butein [82] Catechin [83] Emodin [67] Epichatechin [83] Epigallocatechin gallate [84] Flavonoids [83] Pinosylvin [77] Quercetin [67] Resveratrol [75,76,77,85,86,87] Rhein [67] *trans*- stilbene *cis*-Stilbene [24,67] Quercetin, apigenin, emodin, rhein, or *cis*-stilbene plus chlorambucil, cisplatin or cyclophosphamide [71] Quercetin, apigenin, emodin, rhein, or *cis*-Stilbene plus methotrexate, 6-mercaptopurine or 5-fluorouracil [23] Quercetin, apigenin, emodin, rhein, or *cis*-Stilbene plus doxorubicin or etoposide [24,67]
	MOLT-3	Acute lymphocytic leukaemia	Human	ATCC	Delphinidin [68] Quercetin [68]
	MOLT-4	Acute lymphocytic leukaemia	Human	ATCC	Pinosylvin [77] Quercetin [88,89] Resveratrol [76,77,90] Quercetin plus ellagic acid [88]
	MT-4	Acute lymphoblastic leukaemia	Human	Fernandez et al. 2019 [91]	Butein [92]
	Nalm-6	Acute lymphoblastic leukaemia	Human	ATCC	Resveratrol [66,75,76]
	REH	Acute lymphoblastic leukaemia	Human	ATCC	Resveratrol [75,76]
	RS4;11	Acute lymphoblastic leukaemia	Human	ATCC	Resveratrol [75,76]
	SEM	Acute lymphoblastic leukaemia	Human	Greil et al. 1994 [93]	Resveratrol [75,76]
	SUP-B15	Acute lymphocytic leukaemia	Human	ATCC	Resveratrol [87]
	TL-Oml	Acute lymphoblastic leukaemia	Human	Sugamura et al. 1984 [94]	Butein [92]
	WSU-CLL	Chronic lymphocytic leukaemia	Human	Mohammad et al. 1996 [95]	Resveratrol [66,96] e-Viniferin plus Vineatrol [80]
	L1210	Lymphocytic leukaemia	Mouse	ATCC	Curcumin [97] Gallic acid [97] Quercetin [97,98] Tannic acid [97] Resveratrol [99]
**MYELOID** **LEUKAEMIA**	AML-2/DX30, AML-2/DX100 AML-2/DX300	Doxorubicin resistant acute myeloid leukaemia cell lines	Human	Kweon et al. 2010 [100]	Resveratrol [100]
	AML-2/WT	Ara-C resistant acute myeloid leukaemia	Human	Song et al. 2009 [101]	Resveratrol [100]
	HL-60	Acute promyelocytic leukaemia	Human	ATCC	Apigenin [102,103] Butein [104] Chrysin [103] Carnosic acid [105,106] Curcumin [106,107,108,109] Delphinidin [68] Ellagic acid [66] Epigallocatechin gallate [110] Flavonoids [111] Gallic acid [66] Luteolin [112] Punicalagin [68] Quercetin [68,108,109,113,114] Resveratrol [76,90,96,115,116,117,118,119] Silibinin [107] (-)-Vitisin [120] Xanthohumol [121] Curcumin plus silibinin or carnosic acid [107] Ellagic acid plus all-trans-retinoic acid [122] Gallic acid, ellagic acid plus all-trans retinoic acid [122] Green or black tea [123]
	K562	Chronic myelogenous leukaemia	Human	ATCC	*Allium cepa L*. (PEAL) polyphenols [124] Butein [82,104] Curcumin [125] Epigallocatechin gallate [126,127] Flavopiridol [128] Quercetin [129] Resveratrol [76,81,96,130] Green or black tea [123] Resveratrol plus bestatin [130] Woodfordin I extract (high in tannins) [123]
	K562/ADR	Adriamycin-resistant chronic myeloid leukaemia cell line	Human	Tsuruo et al. 1986 [131]	Resveratrol plus bestatin [130]
	IM-S and IM-R K562	Imatinib-sensitive and resistant chronic myelogenous leukaemia	Human	Grosso et al. 2009 [132]	Resveratrol [133]
	Kasumi-1	Acute myeloblastic leukaemia	Human	ATCC	Resveratrol [87]
	KCL22	Chronic myeloid leukaemia	Human	ATCC	Resveratrol [96]
	KG-1a	Acute myelogenous leukaemia	Human	ATCC	Aloe-emodin [67] Apigenin [23,67] Carnosic acid [106,107] Curcumin [106,107] Quercetin [23,67] Emodin [23,67] Rhein [23,67] Resveratrol [134,135] Silibinin [107] *cis*-Stilbene [23,67] trans-Stilbene [67] Curcumin plus silibinin or carnosic acid [107] Quercetin, apigenin plus doxorubicin or etoposide [24] Quercetin, apigenin plus *cis*-platin, cyclophosphamide or chlorambucil [71]
	LAMA84	Chronic myeloid leukaemia	Human	ATCC	Flavopiridol [128]
	MV4:11	Biphenotypic B myelomonocytic myeloid leukaemia	Human	ATCC	Resveratrol [75,76]
	NB4	Acute promyelocytic leukaemia	Human	Lanotte et al. 1991 [136]	Carnosic acid [106] Curcumin [106] Epigallocatechin gallate [110] Genistein [114,115] Quercetin [114] Resveratrol [115]
	OCI/AML3	Acute myeloid leukaemia	Human	Quentmeier et al. 2005 [137]	Resveratrol [76]
	OCIM2	Acute myeloid leukaemia	Human	Papayannopoulou et al. 1988 [138]	Resveratrol [76]
	SHI-1	Acute monocytic leukaemia	Human	Chen et al. 2005 [139]	Curcumin [140]
	THP-1	Acute monocytic leukaemia	Human	ATCC	*Allium cepa L*. (PEAL) polyphenols [141] Aloe-emodin [67] Apigenin [67] Butein (104] Delphinidin [68] Emodin [67] Flavonoids [69] Genistein [114] Pinosylvin [142] Punicalagin [68] Quercetin [67,68,114] Resveratrol [96,142] Rhein [67] *cis*-stilbene [67] *trans*-Stilbene [67] Quercetin, apigenin, emodin, rhein, or *cis*-stilbene plus chlorambucil, cisplatin or cyclophosphamide [71] Quercetin, apigenin, emodin, rhein, or *cis*-Stilbene plus methotrexate, 6-mercaptopurine or 5-fluorouracil [23] Quercetin, apigenin, emodin, rhein, or *cis*-Stilbene plus doxorubicin or etoposide [24]
	C1498 (TIB-49)	Acute myeloid leukaemia	Mouse	ATCC	Carnosic acid [106] Curcumin [106]
**MYELOMA**	MM144	Plasma cell myeloma	Human	Díaz-Rodríguez et al. 2011 [143]	Resveratrol [86]
	MM1S	Immunoglobulin A lambda myeloma	Human	ATCC	Resveratrol [86]
	U266	Myeloma; plasmacytoma	Human	ATCC	Resveratrol [86]
**LYMPHOMA**	Raji	Burkitt’s lymphoma cell line	Human	ATCC	Epigallocatechin-gallate [144]
	U-937	Histiocytic lymphoma	Human	ATCC	Butein [82,104] Carnosic acid [105,106] Curcumin [106] Flavonoids [145] Guggulsterone [146] Icariside II [147] Piceatannol [148] Pinosylvin [142] Quercetin [113,124] Resveratrol [96,115,142,149] Polyphenols extracted from *lyophilized Lonicera japonica* (PELJ) [150]
	BKS-2	B lymphoma cell line	Mouse	Udhayakumar et al. 1989 [151]	Curcumin [152]
	WEHI-231	B lymphoma cell line	Mouse	ATCC	Curcumin [152]
**HEREDITARY SPHEROCYTSIS**	WIL2-NS	B lymphocyte	Human	ATCC	Resveratrol [153]

## Abbreviations

Acute myeloid leukaemia (AML), Acute lymphoblastic leukaemia (ALL), Chronic myelogenous leukaemia (CML), Chronic lymphocytic leukaemia (CLL), Cyclooxygenases (COX-) 1 and (COX-) 2, Mitochondrial membrane potential (MMP), LRP (lung resistance protein), Glutathione (GSH), Signal transducer and activator of transcription 3 (STAT3), Granulocyte colony-stimulating factor (G-CSF), Suppressor of cytokine signaling (SOCS), inhibitor of activated STAT3 (PIAS3), lymphotoxin β (LTβ), receptor activator nuclear factor ligand (RANKL), IκB kinase (IKK), B-cell-activating factor (BAFF), Protein kinase B (Akt), Extracellular signal-regulated kinases (ERKs), Tumour necrosis factor-α (TNFα), inhibitor of NF-κB (IκBα), Mitogen-activated protein kinase (MAPKs), Nuclear factor κB (NF-κB), Phosphoinositide 3-kinase (PI3K), Mammalian target of rapamycin (mTOR), Interleukin-1 (IL-1), Interleukin-6 (IL-6), Interleukin-17 (IL-17), Interleukin-23 (IL-23), CC-chemokine ligand 2 (CCL2), Reactive oxygen species (ROS), Heme oxygenase-1 (HO-1), Glutathione S-transferases (GST), Adenosine triphosphate (ATP), Cyclin dependent kinases 1 (CDK1), Cyclin dependent kinases 2 (CDK2), Cyclin dependent kinases 4 (CDK4), Cyclin dependent kinases 6 (CDK6), Cyclin-dependent Kinase Inhibitor (p21Cip/Waf1), Cyclin-dependent kinase inhibitor (p27Kip1), mitogen-activated protein (MAP), B-cell lymphoma 2 protein (Bcl-2), B-cell lymphoma-extra-large (Bcl-xL), X-linked inhibitor of apoptosis protein (XIAP), Cellular Inhibitor of Apoptosis Protein 1(cIAP-1), Cellular Inhibitor of Apoptosis Protein 2 (cIAP-2), Myeloid cell leukaemia 1 (Mcl-1), Cysteine aspartic acid specific protease 3, 8 and 9 (Caspase 3, 8 and 9), APO-l/CD95/tumour necrosis factor superfamily 6 (TNFRSF6)/APT-1(Fas), Fas Ligand (FasL), Tumour necrosis factor receptor (TNF), Tumour necrosis factor-alpha (TNF-α), Fas-associated Protein with Death Domain (FADD), Death receptor 4 and 5 (DR4 and DR 5), Bcl-2-like protein (Bim), Bcl-2-associated X protein (Bax), Bcl-2-antagonist/killer (Bak), The Bcl-2 associated agonist of cell death (Bad), BH3 interacting-domain death agonist (Bid), Poly (ADP-ribose) polymerase (PARP), Light chain I and II (LC3-I and LC3-II), Permeability glycoprotein (P-gp), ATP Binding Cassette Subfamily C Member 1 (ABCC1), Unc-51 Like Autophagy Activating Kinase 1 (ULK1), and Atg17, WD-repeat domain phosphoinositide-interacting proteins (WIPIs) and double FYVE domain-containing protein 1 (DFCP1), AMP-activated kinase (AMPK), Growth Arrest and DNA Damage (GADD45), Second mitochondria-derived activator of caspase a mitochondrial protein (SMAC), Mitochondrial serine protease (Omi), Apoptotic protease-activating factor-1 (APAF-1), Lymphotoxin-alpha (LT-α), TNF-like protein-1A (TL1A), Apo2L/TNF-related apoptosis-inducing ligand (TRAIL), TNF receptor-associated death domain (TRADD), Death-inducing signalling complex (DISC), FADD-like IL-1β-converting enzyme-inhibitory protein (c-FLIP), SMAC (Second mitochondria-derived activator of caspase)/DIABLO (Direct Inhibitor of Apoptosis-Binding protein with LOw pI) (SMAC/DIABLO), Chemokine (C-C motif) ligand 2 (CCL2), The inhibitor of kappa B kinase epsilon (IKBKE), Sequestosome 1/ubiquitin-binding protein p62 (SQSTM1)/p62, (Beclin 1), vacuolar protein sorting 34 (VPS34), class III phosphatidylinositol 3-kinase (PIK3C3), autophagy-related genes (ATG genes), Activating Molecule in Beclin1-Regulated Autophagy (AMBRA1), DNA damage-regulated autophagy modulator 1 (DRAM1).
